# Loss-of-function Mutations K11E or E271K Lead to Novel Tumor Suppression, Implicate Nucleolar Helicase DDX24 Oncogenicity

**DOI:** 10.7150/ijms.67840

**Published:** 2022-03-14

**Authors:** Xinglin Li, Xiaoyun Chen, Jiebing Gao, Jianzhong Xian, Zhijun Li, Lei Bi, Min Yang, Shuai Yang, Hongjun Jin, Hong Shan

**Affiliations:** 1 Guangdong Provincial Key Laboratory of Biomedical Imaging and Guangdong Provincial Engineering Research Center of Molecular Imaging, The Fifth Affiliated Hospital, Sun Yat-sen University, Zhuhai, Guangdong Province 519000, China; 2 Department of Ultrasound, The First Affiliated Hospital of Shenzhen University Health Science Center, Shenzhen Second People's Hospital, Shenzhen, Guangdong Province 518000, China; 3 Department of Radiology, Zhongshan Affiliated Hospital, Guangzhou University of Chinese Medicine, Zhongshan, Guangdong Province 528400, China; 4 Department of Radiology, The Fifth Affiliated Hospital, Sun Yat-sen University, Zhuhai, Guangdong Province 519000, China; 5 Department of Ultrasound, The Fifth Affiliated Hospital, Sun Yat-sen University, Zhuhai, Guangdong Province 519000, China; 6 Center of Oncology, The Fifth Affiliated Hospital, Sun Yat-sen University, Zhuhai, Guangdong Province 519000, China; 7 Department of Interventional Medicine, The Fifth Affiliated Hospital, Sun Yat-sen University, Zhuhai, Guangdong Province 519000, China

**Keywords:** Nucleolar, mutation, loss-of-function, DDX24

## Abstract

**Purpose:** Mutations (K11E or E271K) of DEAD-box RNA helicase 24 (DDX24) were related to multi-organ venous lymphatic malformation syndrome (MOVLD). However, the relationship between these mutations and DDX24-function still remains unknown. Understanding whether K11E and E271K cause “loss-of-function” or “gain-of-function” for DDX24 is significant for related diseases. DDX24 was reported to be related to tumors closely, thus this study aims to explore how K11E and E271K affect DDX24-function in tumor proliferation.

**Methods:** Cell lines stably expressing wild-type DDX24, K11E-DDX24, E271K-DDX24, along with vector only based on Chinese hamster ovary cells (CHO) and Balb/c tumor-bearing mice models were constructed. Then immunofluorescence staining, proliferation assay and colony formation assay *in vitro* and ^18^F-FDG PET/CT-scan were performed. Finally, the tumor tissues were collected to perform transcriptome sequencing to predict the potential mechanism.

**Results:** Contrasted with CHO-WT-DDX24, CHO-K11E-DDX24 or CHO-E271K-DDX24 showed a decreased number of nucleoli, a slower proliferation rate and a lower colony formation rate significantly. Moreover, mice, inoculated with CHO-K11E-DDX24 or CHO-E271K-DDX24 cells, showed lower tumor formation rate, slower tumor growth rate, better prognosis, reduced standard uptake value and K_i_ of glucose in subcutaneous tumors. Sequencing indicated CHO-K11E-DDX24 or CHO-E271K-DDX24 caused increasing expression of TNF or chemokines and alteration in immune-related signal pathways.

**Conclusion:** K11E or E271K mutation could lead to “loss-of-function” of DDX24 in cell proliferation and tumor bearing mice, which may be acted by non-specific immune killing to inhibit tumor growth.

## Introduction

DEAD-box RNA helicases, named because of the characteristic Asp-Glu-Ala-Asp (DEAD) in the conserved motif, referred to as DDX, are the largest family in super family 2 helicases 1. The helicase core region of DDX consists of two RecA-like domains with at least twelve conserved motifs, serving binding sites for ATP and RNA. The N-terminal and C-terminal domains, flanking the helicase core, confer diverse functions on DDX by interacting with other protein and RNAs (Figure S1A). The Q motif, together with motif I (or Walker A motif), motif II (or Walker B motif), and motif VI, is required for ATP binding and hydrolysis. Motifs Ia, Ib, Ic, IV, IVa and V are characterized less well but may be involved in RNA binding. In addition, motif III and Va may communicate ATPase and unwinding activities between ATP binding and RNA binding sites 2-4. DDX mainly is participated in the regulation of RNA metabolism such as RNA export, RNA decay, RNA storage and ribosome biogenesis. Besides, DDX possesses a crucial role in transcriptional regulation of gene expression. Increasing researches suggested that DDX were highly multifunctional with positive character in carcinogenesis. For example, DDX27 was reported to enhance NF-κB signaling by increasing nucleophosmin-1 (NPM1) and NF-κB-p65 interaction in colorectal cancer 5. DDX21 was reported as a positive regulator of polymerase-1 and potentiated cell growth in breast cancer 6. DDX5 was regarded as a poor prognosis predictor for triple-negative breast cancer patients, because it promoted cancer cells proliferation by interacting with fos-related antigen 1 7.

As a member of DDX family, DDX24 plays a critical role for the ribosome biogenesis 4. In terms of immune function, previous studies showed that DDX24 inhibited the function of RIG-I-like receptors specifically to participate in the regulation of innate immune 8. Moreover, DDX24 could promote the proliferation of HIV-1 virus 9. In terms of tumor function, DDX24 was identified as a drug target in cancer therapeutics by screening of a shRNA library 10. Yamauchi T, Shi D and their colleagues reported that the depletion of DDX24 might block p53 degradation by attenuating MDM2-mediated ubiquitylation or activating p300-p53 axis 11, 12. In addition, previous study has figured out the point mutations of *DDX24*, K11E and E271K, were closely associated with multi-organ venous and lymphatic defect syndrome (MOVLD), in which *DDX24* gene was confirmed to be involved in VEGF signaling and cell migration pathways 13. The 11aa and 271aa sites are located at the amino domain and the core domain of DDX24, respectively. In addition, both 11aa and 271aa sites are highly conserved across distinct species (Figure S1B). Mutations in highly conserved regions are likely to produce important biological function changes 14, 15. However, whether these mutations work as “loss of function” or “gain of function” remains unknown. DDX24 seemed to be a potential oncogene, thus our study here investigated how K11E and E271K affect the function of DDX24 in tumorigenesis in cell and in animal model.

DDX24 is generally expressed in human tissues and cells, therefore human cells were not suitable to build DDX24 point mutation model studies. Chinese hamster ovary cell (CHO) line, without detection of DDX24 expression, is an ideal cell to contrast wild-type and mutant DDX24. Moreover, CHO cells were widely used in the evaluation of gene functions by transfecting the target gene *in vitro* and *in vivo*, especially when long-term and stable gene expression was needed 16. Thus, cell lines stably expressing wild-type DDX24, K11E-DDX24 and E271K-DDX24 based on CHO cells were constructed to explore the differences between wild-type DDX24 and mutant DDX24 in tumor proliferation. Here, we report our study regarding K11E or E271K DDX24 functions of oncogenicity using the CHO cells and tumor bearing mice model.

## Methods

### Cell lines and cell culture

Chinese hamster ovary (CHO)-K1 cell line was obtained from Institute of Biophysics, Chinese Academy of Sciences. CHO-K1 cells were cultured in DMEM/F12 medium with 10% fetal bovine serum and 1% penicillin-streptomycin at 37 ℃ in a humidified atmosphere containing 5% CO_2_. Human MHCC-97L (liver cancer), KYSE410 (esophagus squamous carcinoma), PC3 (prostate carcinoma) and HeLa (cervical cancer) cell lines were obtained from Sun Yat-sen University Medical Research Cell Banks and were cultured in complete DMEM or 1640 medium.

### Establishment of monoclonal stably transfected cells

The following plasmids were synthesized by Yingrun Biotechnology Company (Hunan, China) and the sequence of corresponding primer was shown in Table S1. CHO-K1 cells were transfected with pmCherry-N1-WT-DDX24, pmCherry-N1-K11E-DDX24, pmCherry-N1-E271K-DDX24, along with pmCherry-N1 plasmid, respectively (Figure S2A, 2B) by using Lipofectamine^TM^ 3000 (L3000015, Thermo Fisher) and Opti-MEM^®^ Reduced Serum Medium (31985070, Thermo Fisher), and then were cultured in a 96-well plate (100 cells/10mL) with medium containing 1500 μg/mL G418 (G8160-1, Solarbio). After two weeks, single cell clone with red-fluorescent was selected for expanding gradually to obtain a monoclonal cell line. Sequencing analyses of CHO-WT-DDX24, CHO-K11E-DDX24 and CHO-E271K-DDX24 cell lines (Figure S2C) showed that no mutations were observed in WT, while 31nt site mutation (A to G) was observed in CHO-K11E-DDX24 and 811nt site mutation (G to A) was observed in CHO-E271K-DDX24.

### Quantitative real-time PCR

Total RNA was extracted from cells following the protocol of Total RNA Kit (R6834-02, Omega), and was reversely transcribed into cDNA using cDNA Synthesis Kit (M1681, Thermo Fisher). Transcript levels were detected by using SYBR Green Master Mix (A25742, Thermo Fisher) under a QuantStudio 7 Flex System (Thermo Fisher, USA). The following primers were used: DDX24, forward CCTGCCATCCGTGACAAACT, reverse AGGTGGTGCTTCGGTGTTAC; β-actin, forward CCTGGCACCCAGCACAAT, reverse GGGCCGGACTCGTCATACT (sequences from 5' to 3').

### Western blot analysis

Total proteins from cells were extracted using RIPA lysis buffer (89900, Thermo Fisher) supplemental with Protease Inhibitor Cocktail (CW2200S, CWBIO). After quantification by BCA assay (P0010, Beyotime), equal amounts (30 μg) of proteins were electrophoresed on SDS-PAGE gels (M00661, GenScript) and then were transferred to PVDF membranes (ISEQ00010, Merck Millipore). Blots were incubated with the primary antibodies including DDX24 (ab70463, Abcam) or GADPH (60004, Proteintech). Bands were visualized under a Chemiluminescence Reaction System (Bio-Rad, USA) following manufacturer's recommendations.

### Immunofluorescence (IF) staining

IF staining was performed to confirm the nucleolus location of DDX24 in the constructed cells. Cells were fixed with 4% paraformaldehyde (DF0135, Leagene) for 30 min, then were incubated with 0.5% Triton X-100 (1139ML500, Biofroxx) for 20 min and with 5% goat serum (16210064, GIBCO) at room temperature for 1 h in turn. Subsequently, cells were incubated with 5 μg/mL NPM1 antibody (32-5200, Invitrogen) overnight at 4 ℃ and then with secondary antibody conjugating Alexa Fluor® 488 (ab150125, Abcam) for 1 h at room temperature protected from light. At last, cells were antifaded by mounting medium with DAPI (H-1200, Vector) and were photographed under a Confocal Microscope (Zeiss, Germany). Cells were washed three times with PBST at every solution removal.

### Cell proliferation assay

CHO-Vector, CHO-WT-DDX24, CHO-K11E-DDX24, CHO-E271K-DDX24 (800 cells/well), MHCC-97L, KYSE410, PC3 and HeLa cells (1200 cells/well) were inoculated in 96-well plates. After 24, 48, 72, 96 h cultured in an incubator, cell counting kit-8 (CCK-8) solution (CK04, Dojindo) was added to each well for 2 h. The absorbance at 450 nm was determined under a Microplate Reader (Synergy HTX, BioTek, USA) and then the cell growth curves were depicted basing on the OD values at indicated time points.

### Colony formation assay

After CHO-Vector, CHO-WT-DDX24, CHO-K11E-DDX24, CHO-E271K-DDX24 cells were inoculated (1000 cells/well) in 6-well plates and were cultured in complete medium for 10 days, cells were fixed using 4% paraformaldehyde for 30 min and then were stained using crystal violet (G1062, Solarbio) for 30 min at room temperature. Then the colonies were pictured under the Chemiluminescence Reaction System (Bio-Rad, USA) and were counted by Image J (NIH).

### Tumorigenicity assay *in vivo*

All animal experiments were performed according to the institutional ethical guidelines for animal experiments of The Fifth Affiliated Hospital of Sun Yat-sen University. Balb/c nude mice 4 ~ 6 weeks old were purchased from Medical Animal Experiment Center of Guangdong Province. Mice were randomly divided into four groups and then were injected subcutaneously with CHO-Vector, CHO-WT-DDX24, CHO-K11E-DDX24 and CHO-E271K-DDX24 cells at a dose of 5 x 10^6^ in the left flanks, respectively. Tumor growth was monitored by measuring the volume with a vernier caliper. For the tumor-free survival curves, mice were considered tumor-free until tumors were visible or palpable (> 0.5 cm in diameter).

### PET/CT ^18^F-FDG imaging

Mouse PET/CT scan was carried out when the tumor volume reached 200 mm^3^ using a NanoScan PET/CT system (Mediso, Hungary). Mice were anesthetized with isoflurane gas (2.5% for induction and 1.5% for maintenance). ^18^F-FDG (4.44 ~ 5.55 MBq) was administered intravenously via the tail vein. An emission scan of 60 min was acquired for all mice. The PET data were acquired in list mode and were reconstructed into 25 timeframes (10 x 3 s, 3 x 10 s, 4 x 60 s, 5 x 300 s, 3 x 600 s). The evaluation of dynamic PET data including standardized uptake value (SUV) was performed using the software of Carimas (Version 2.9) (Turku PET Center, Finland). The influx rate constant (K_i_) of tumor was calculated using Patlak analysis by applying tumor time-activity curve (TAC) data to the Matlab software (Version 2014a) as we previously reported 17.

### Immunohistochemistry (IHC) staining

The tumors were dissected and fixed in 10% buffered formalin. The tissue samples were paraffin-embedded and sectioned at 5 μm thickness. The slices were deparaffinized in xylene and rehydrated in graded alcohols. Briefly, after blocked with 5% goat serum for 60 min at room temperature, slices were incubated with primary antibodies (1: 200 dilution) anti-DDX24 (PA5-51721, Thermo Fisher) at 4 ℃ overnight, followed by the corresponding secondary antibody sheep anti-rabbit IgG polymer (MXB Biotechnologies, China) for 30 min at room temperature. Subsequently, the slices were treated with liquid 3,3′-diaminobenzidine (MXB Biotechnologies) and counterstained with hematoxylin. Finally, all the sections were scanned under a Pannoramic 250 Flash (3DHISTECH, Hungary).

### RNA-sequencing (RNA-Seq)

To further explore the underlying molecular mechanisms of WT-DDX24 expression on promoting proliferation and K11E-DDX24 expression on suppressing tumorigenicity, the RNA-seq of tumors from Balb/c-nu mice of four groups, were used to estimate the transcriptome influence of wild-type or mutant DDX24 expressing cells on the surrounding microenvironment. Thus, 15 samples (4 replicates for Vector, 5 replicates for WT, 2 replicates for K11E and 4 replicates for E271K) were sequenced. Finally, the datasets were generated on BGIseq500 platform (BGI, Guangdong, China).

* P* value was corrected to *Q* value by differentially expressed genes (DEGs**)** sequencing 18. Genes with fold change ≥ 2 and *Q* value ≤ 0.001 were screened as significantly DEGs. To guarantee the contrast, we selected their overlapped DEGs from our datasets. To further identify related pathways and genes, we performed both Kyoto Encyclopedia of Genes and Genomes (KEGG) pathway enrichment and Gene Ontology (GO) term enrichment. Moreover, Gene Set Enrichment Analysis (GSEA) was also performed on all genes in each group to avoid screening genes for false negatives at thresholds. In addition, clustered heatmaps of gene expression were obtained by running the R language.

### Statistical analysis

Statistical analyses were performed through GraphPad Prism (Version 8). Quantitative data were shown as mean ± standard deviation from at least three independent experiments. If data were normally distributed, then unpaired T test or One-way ANOVA tests (Tukey multiple comparisons test, Dunnett multiple comparisons test or Holm-Sidak multiple comparisons test) was used for *p* value calculation. Otherwise, Kruskal Wallis test was used for multiple comparisons. Chi-square (and Fisher exact) test was used for tumor formation rate comparisons. Log-rank (Mantel-Cox) test was used for Kaplan-Meier tumor-free survival comparisons.* P* value, adjusted by test, less than 0.05 was considered statistically significant, where * indicated *p* < 0.05, ** indicated *p* < 0.01, *** indicated *p* < 0.001, respectively.

## Results

### The DDX24 expression was verified in stably transfected cell lines

**Figure 1:**
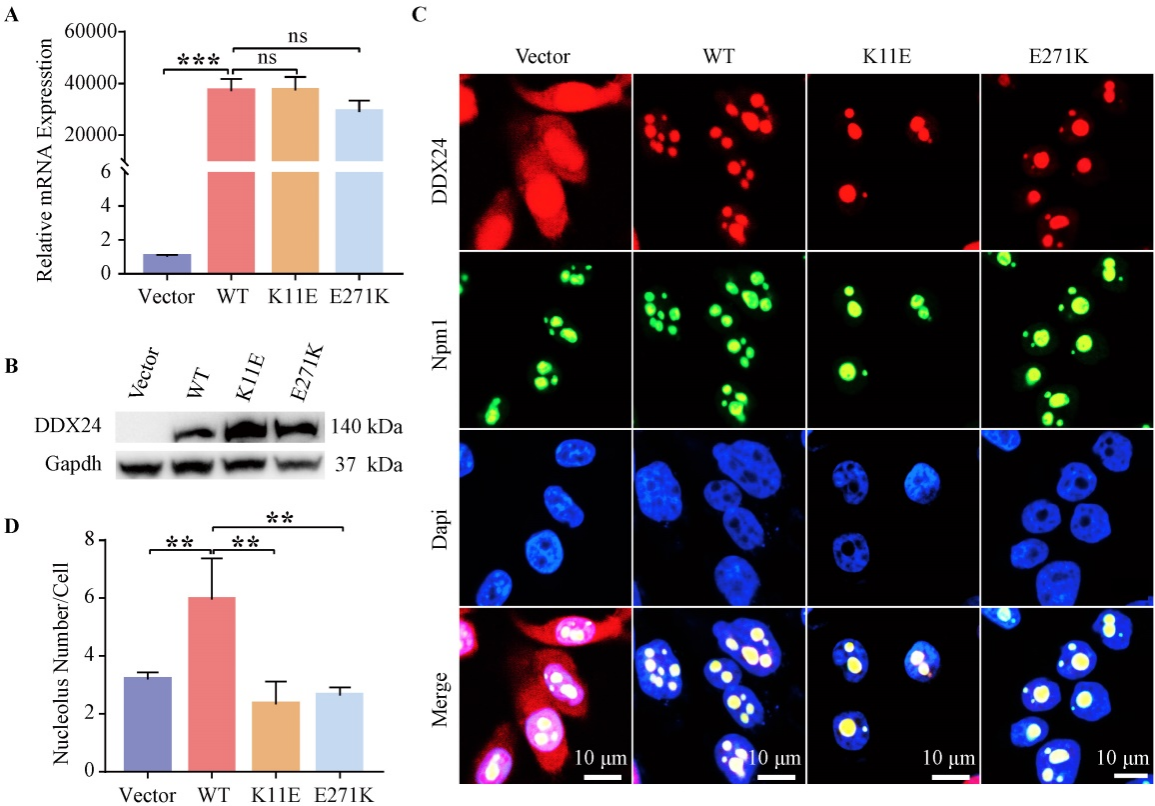
** Detection of DDX24 expression in constructed CHO cell lines. (A)** Relative mRNA levels of DDX24 in constructed CHO cell lines were detected by quantitative real-time PCR. *P* values were calculated with Tukey multiple comparisons test. ***, *p* < 0.001; ns, no significance. **(B)** Detection of DDX24 expression in the constructed CHO cell lines by Western blot.** (C)** Representative images demonstrating the nucleolar localization of DDX24 (co-locating with Npm1) in the cell lines under a confocal microscope. Nuclei were stained with Dapi (blue). Scale bars, 10 μm. **(D)** Quantitative analysis of nucleoli number in cell lines. *P* values were calculated with Dunnett multiple comparisons test. **, *p* < 0.01; Vector *vs.* WT, *p* = 0.0024; WT *vs.* K11E, *p* = 0.0014; WT *vs.* E271K, *p* = 0.0017.

Constructed cell lines exhibited high transcriptional level (up to 20,000 times) of *DDX24* except CHO-Vector, as tested by qPCR (Figure 1A). Western blot further corroborated DDX24 expression in the constructed cells compared to the empty plasmid (Figure 1B). The 11aa and 271aa sites of DDX24 in stably transfected cell lines were also verified by using cell-sequencing (Figure S1C).

### The number of nucleoli in CHO-K11E-DDX24 or CHO-E271K-DDX24 cells was decreased

IF staining showed wild-type and point mutant DDX24 were both co-located with the nucleolar protein Npm1 (Figure 1C). Besides, the number of nucleoli was measured (Figure 1D), which revealed the number of nucleoli both decreased notably in CHO-K11E-DDX24 (2.33 ± 0.78) (*p* = 0.0014) and CHO-E271K-DDX24 (2.94 ± 0.59) (*p* = 0.017) compared with CHO-WT-DDX24 (5.95 ± 1.42).

### The proliferation and colony formation in CHO-K11E-DDX24 and CHO-E271K-DDX24 were suppressed

**Figure 2:**
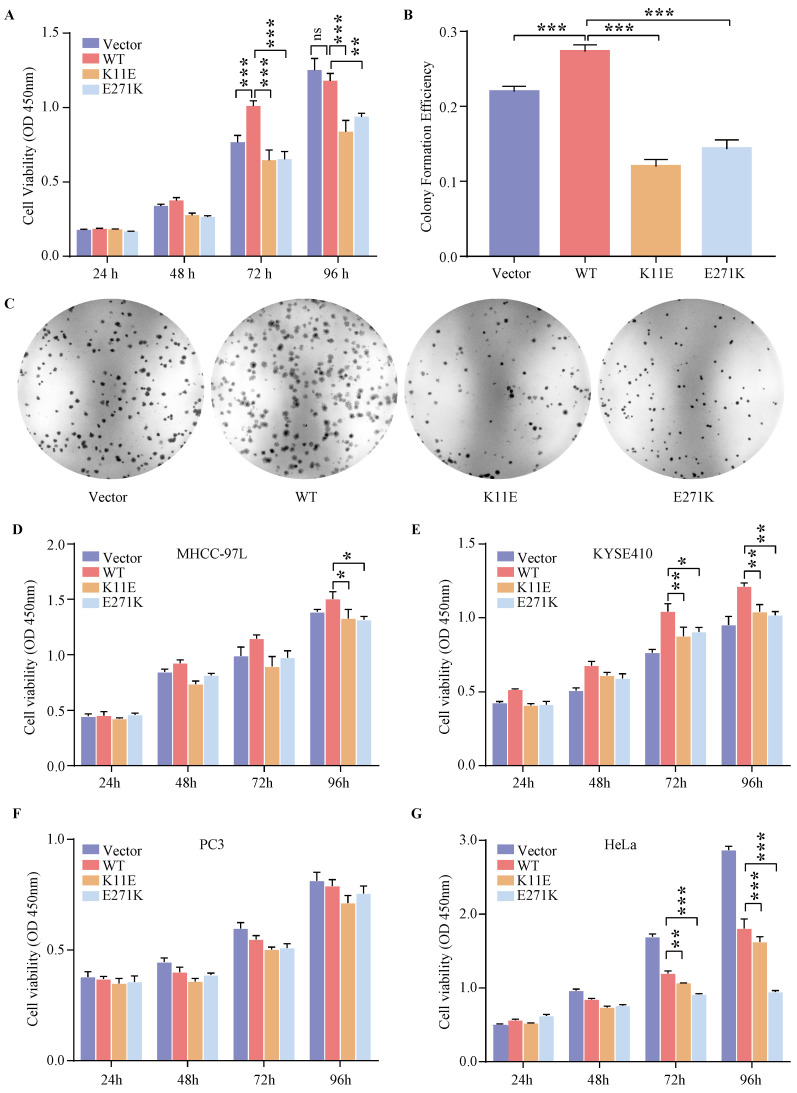
** Expression of DDX24 affected cell proliferation. (A)** Cell proliferation assay in constructed CHO cell lines. *P* values were calculated with Dunnett multiple comparisons test. ***, *p* < 0.001. WT *vs.* K11E: *p* **_72 h_** < 0.0001, *p* **_96 h_** < 0.0001; WT *vs.* E271K: *p* **_72 h_** < 0.0001, *p* **_96 h_** = 0.006.** (B)** Colony formation assay in constructed CHO cell lines. *P* values were calculated with Dunnett multiple comparisons test. ***, *p* < 0.001. **(C)** The colonies were pictured under the Chemiluminescence Reaction System and counted by Image J. Cell Proliferation assay **(D, E, F, G)** in specific tumor cell lines. *P* values were calculated with Tukey multiple comparisons test. *, *p* < 0.05; **, *p* < 0.01; ***, *p* < 0.001. **(D)** WT *vs.* K11E: *p* = 0.0194; WT *vs.* E271K: *p* = 0.0131. **(E)** WT *vs.* K11E: *p* **_72 h_** = 0.0076, *p* **_96 h_** = 0.0042; WT *vs.* E271K: *p* **_72 h_** = 0.0218, *p* **_96 h_** = 0.0019. **(G)** WT *vs.* K11E: *p* **_72 h_** = 0.0022, *p* **_96 h_** < 0.0001; WT *vs.* E271K: *p* < 0.0001.

Expression of WT-DDX24 enhanced the growth of cells at 72 h compared with control vector cells (*p* < 0.0001) (Figure 2A). However, expression of K11E-DDX24 suppressed the growth of cells at 72 and 96 h (*p* < 0.0001). Likewise, expression of E271K-DDX24 inhibited the cell growth at 72 and 96 h (*p* < 0.0001 and *p* = 0.006 respectively). Furthermore, colony formation assay revealed that WT-DDX24 increased the colony forming efficiency (27.30 ± 0.75%, *p* < 0.0001). While the colony formation rates of K11E and E271K were 11.97 ± 0.77% and 14.3 ± 1.00% respectively, which were significantly lower than that of the vector cells (21.97 ± 0.57%, *p* < 0.0001) (Figure 2B, 2C). In addition, after knocking down *DDX24* in CHO-WT-DDX24, cell proliferation and colony formation were inhibited significantly (Figure S3). The effects of point mutation and knockdown were consistent.

### The expression of DDX24 affected the proliferation in human tumor cells

Based on the cancer genome atlas project, we found the expression status of *DDX24* in liver hepatocellular carcinoma (LIHC) and esophageal carcinoma (ESCA) was higher than that in the adjacent normal tissues, while the expression level of *DDX24* in cervical squamous cell carcinoma and endocervical adenocarcinoma (CESC) and prostate adenocarcinoma (PRAD) was lower than that in the adjacent normal tissues (Figure S4A). As shown in Figure S4B, high expression *DDX24* was linked with poor prognosis of overall survival for LIHC (*p* = 0.021). However, low expression of *DDX24* was related to poor overall survival prognosis for CESC (*p* = 0.03) and ESCA (*p* = 0.0059) (Figure S4C). Additionally, the genetic alteration status of *DDX24* in LIHC, ESCA, CESC and PRAD was low (< 4%) (Figure S4D) and neither K11E nor E271K mutation was observed in above cancers (Figure S4E). Transient transfection (verified by qPCR in Figure S5) and CCK8 assay in MHCC-97L, KYSE410, PC3 and HeLa cells were performed. Expression of K11E-DDX24 or E271K-DDX24 suppressed the growth of MHCC-97L cells at 96 h (*p* = 0.0410 and *p* = 0.0192 respectively) compared with WT-DDX24 (Figure 2D). In KYSE410 cells, expression of K11E-DDX24 suppressed the growth at 72 and 96 h (*p* = 0.0076 and *p* = 0.0042 respectively) and expression of E271K-DDX24 suppressed the growth at 72 and 96 h (*p* = 0.0218 and *p* = 0.0019 respectively) compared with WT-DDX24 (Figure 2E). Moreover, there were no significant differences in PC3 cells (Figure 2F). Likewise, expression of K11E-DDX24 suppressed the growth of HeLa cells at 72 and 96 h (*p* = 0.0022 and *p* < 0.0001 respectively) and expression of E271K-DDX24 suppressed the growth at 72 and 96 h (*p* < 0.0001) compared with WT-DDX24 (Figure 2G).

### The tumor growth rate of mice inoculated with CHO-K11E-DDX24 was markedly slowed down

**Figure 3:**
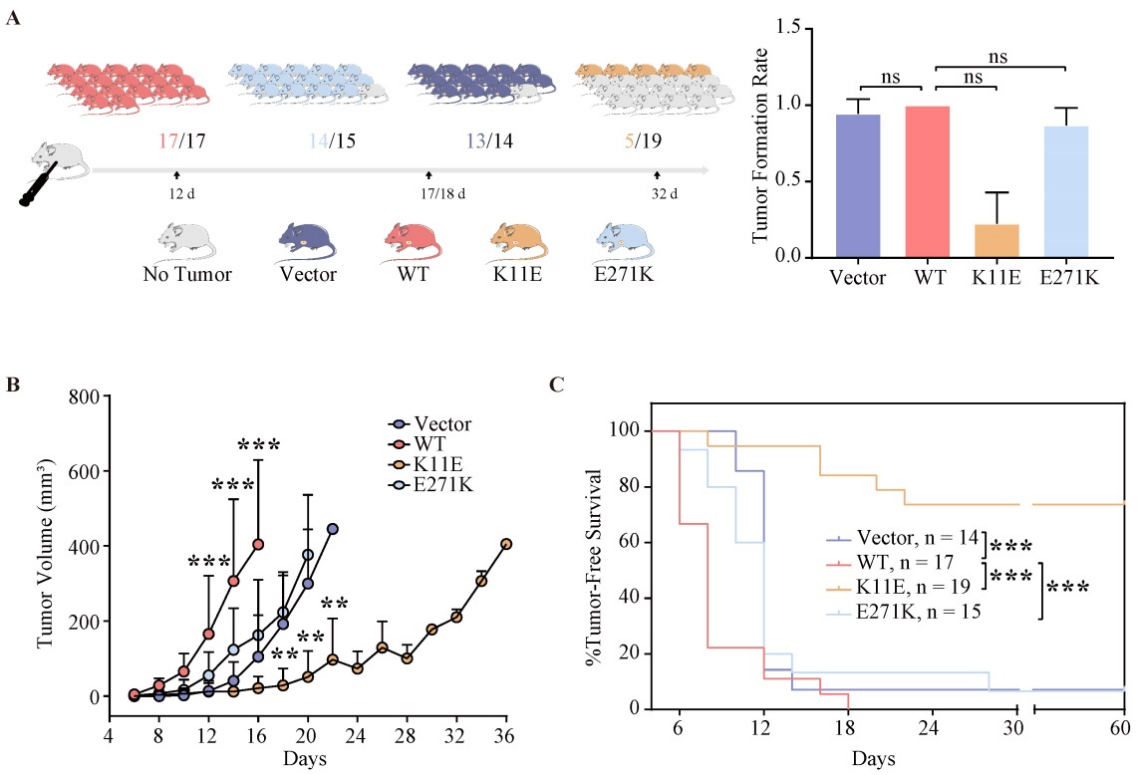
** Wild-type DDX24 enhanced tumorigenicity *in vivo*, while mutant DDX24 inhibited tumor-growth. (A)** The numbers under the gray line segment represented the median day of tumorigenesis and above the gray line segment represented the number of mice with tumor/total number of mice in four independent replicated experiments. Tumor formation rates showed no statistics significance.** (B)** Tumor volume was measured every 2 days after cell transplantation. CHO-Vector group was used as negative control. **(C)** Kaplan-Meier tumor-free survival curves of the mice were shown. *P* values in **(B)** and **(C)** were calculated with Log-rank (Mantel-Cox) test. **, *p* < 0.01; ***, *p* < 0.001.

All mice (17 of 17) that were inoculated with CHO-WT-DDX24 cells developed tumors. 93.3% mice (14 of 15) inoculated with CHO-E271K-DDX24 cells developed tumors, which was nearly equal to CHO-Vector (92.86%, 13 of 14). However, mice inoculated with CHO-K11E-DDX24 cells showed a lower tumor formation rate (only 26.3% mice, 5 of 19) (Figure 3A). The median day of tumor volume reaching 200 mm^3^ in mice inoculated with CHO-WT-DDX24 was 12. While the median day of tumor volume reaching 200 mm^3^ in mice inoculated with CHO-E271K-DDX24 was 17, which was nearly close to CHO-Vector group (18 days). However, the tumor in mice inoculated with CHO-K11E-DDX24 spent 32 days to reaching 200 mm^3^ (Figure 3B). The tumor growth rate of mice inoculated with CHO-K11E-DDX24 was slowed down significantly.

### The survival time of mice inoculated with CHO-K11E-DDX24 was prolonged

The median survival time of mice was 6 ~ 8 days in CHO-WT-DDX24 group, while the CHO-E271K-DDX24 or CHO-Vector groups were 10 ~ 12 days. However, the median survival time of mice inoculated with CHO-K11E-DDX24 was exceed 32 days (Figure 3C). In general, the survival time of mice inoculated with CHO-K11E-DDX24 was significantly prolonged.

### The SUV and K_i_ of tumors formed by CHO-K11E-DDX24 or CHO-E271K-DDX24 were reduced

**Figure 4:**
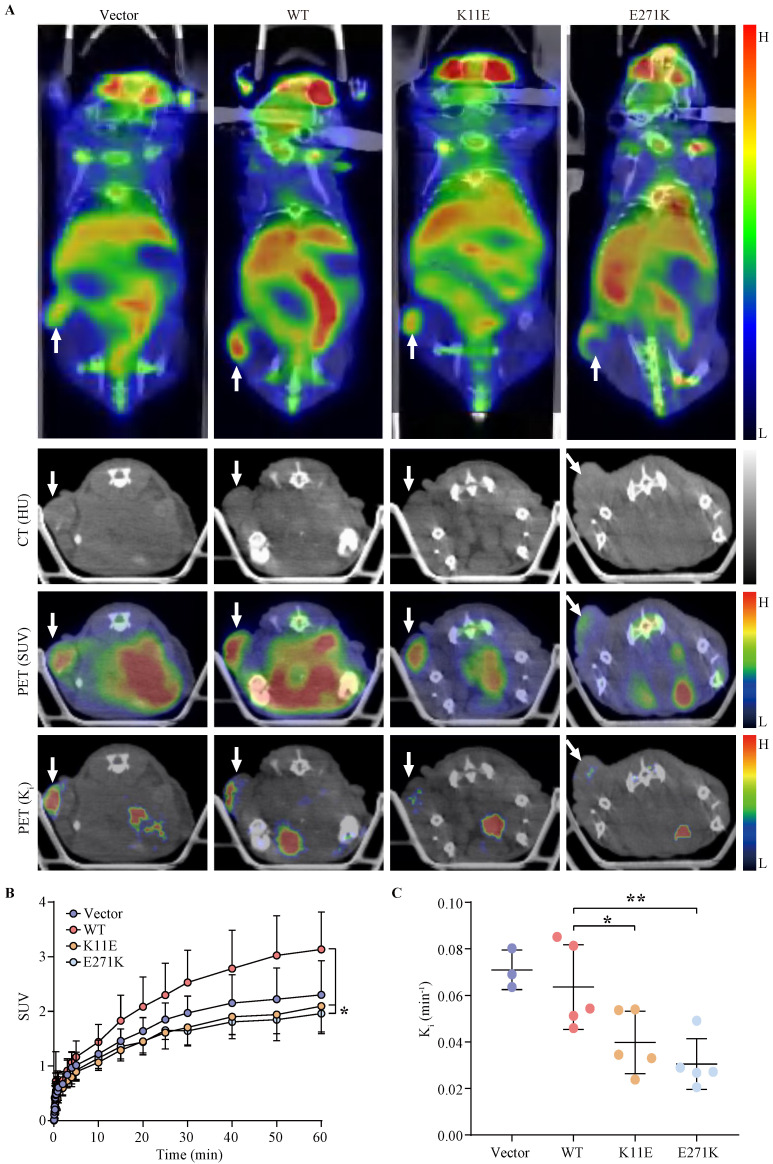
** Loss-of-function K11E or E271K mutation showed decreased glucose utilization rate.** Mice PET/CT scan was scheduled when tumor volume reached 200 mm³. **(A)** Representative transaxial PET/CT images showed similar and intermediate signal in the constructed CHO cells formed tumors (white arrow). The level of signal was represented by a color range from black (low) to red (high). SUV **(B)** and K_i_ **(C)** showed significant difference among the constructed tumors. *P* values in** (B)** were calculated with Tukey multiple comparisons test; *, *p* < 0.05; WT *vs.* K11E, *p* = 0.0410; WT *vs.* E271K, *p* = 0.0192. *P* values in **(C)** were calculated with Holm-Sidak multiple comparisons test. *, *p* < 0.05; **, *p* < 0.01; WT *vs.* K11E, *p* = 0.0484; WT *vs.* E271K, *p* = 0.0099.

Tumor bearing mice were scheduled for dynamic ^18^F-FDG PET/CT scan when the tumor grew up to 200 mm^3^. The TAC of tumors was calculated. Representative PET/CT images were shown in Figure 4A. Higher ^18^F-FDG uptakes of WT-DDX24 group could be observed with corresponding SUV values at 3.13 ± 0.68 (Figure 4B), while the SUV values of K11E (2.10 ± 0.50) and E271K (1.96 ± 0.34) groups were obviously lower (*p* = 0.0410 and *p* = 0.0192 respectively). Similarly, the values of K_i_ from WT-DDX24 group (0.064 ± 0.018 min^-1^) were higher than that of K11E (0.040 ± 0.013 min^-1^) (*p* < 0.05) and E271K (0.030 ± 0.011 min^-1^) groups (*p* = 0.0484 and *p* = 0.0099 respectively) (Figure 4C, 4D), which indicated that K11E or E271K mutation of DDX24 resulted in suppressing the tumor cell proliferation.

### The number of nucleoli in tumor tissue formed by CHO-K11E-DDX24 or CHO-E271K-DDX24 was decreased

DDX24 was highly expressed in tumor tissue formed by CHO-WT-DDX24, CHO-K11E-DDX24 or CHO-E271K-DDX24, except CHO-Vector (Figure S6). At the same magnification, the tumor tissues formed by CHO-WT-DDX24 showed higher density of nucleolus, while the mutant tumor exhibited less density of nucleolus.

### CHO-K11E-DDX24 or CHO-E271K-DDX24 caused increased expression of tumor necrosis factors (TNF) and chemokines, along with alteration in immune-related signal pathways

**Figure 5:**
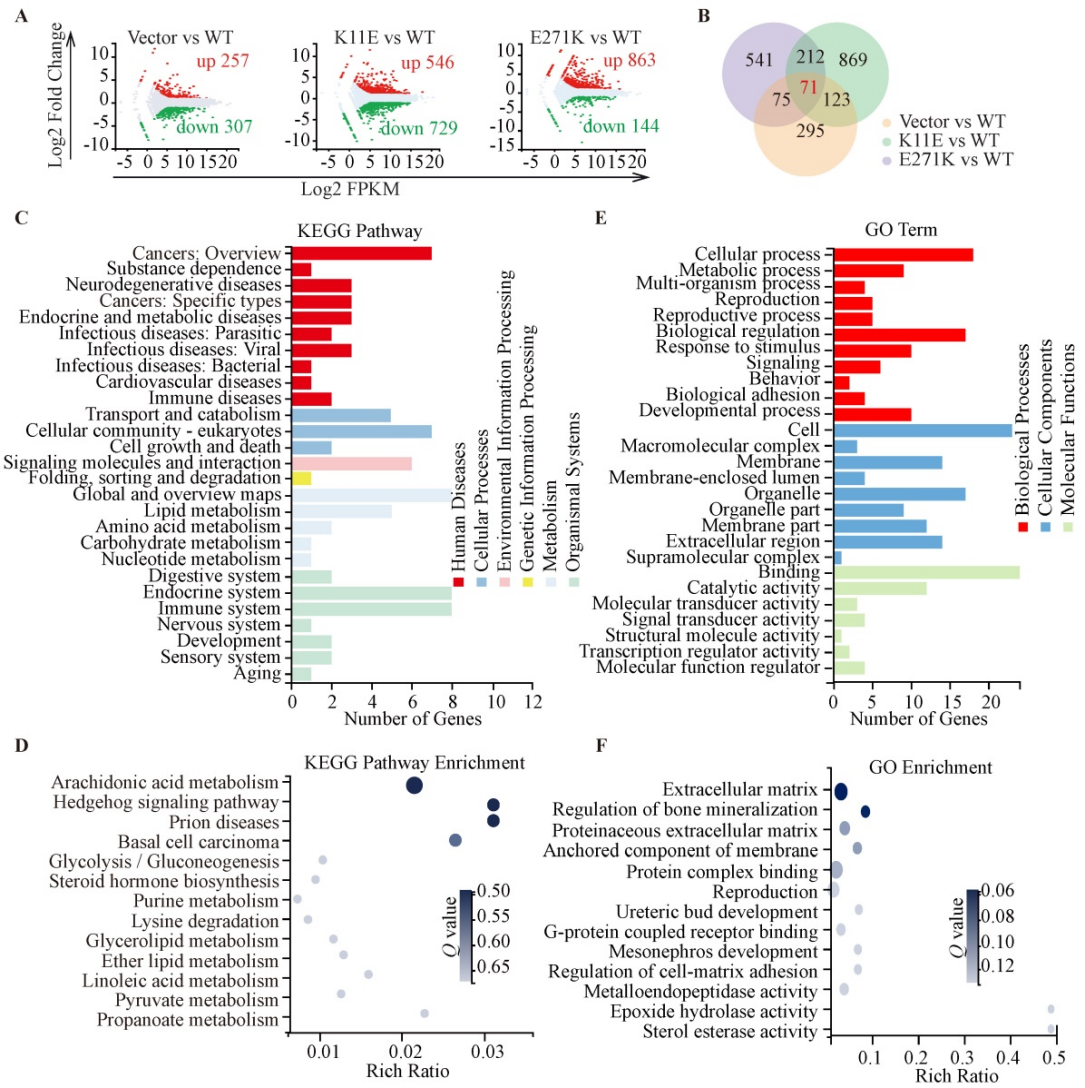
** Sequencing-based transcriptomic analyses. (A)** Red dots represented the up-regulated DEGs, while green dots represented the down-regulated DEGs, and gray dots represented non-DEGs. **(B)** The Venn diagram showed that there were 71 overlapped DEGs in three groups. KEGG pathway classification and enrichment **(C, D)** and GO feature classifications and enrichment** (E, F)**.

**Figure 6:**
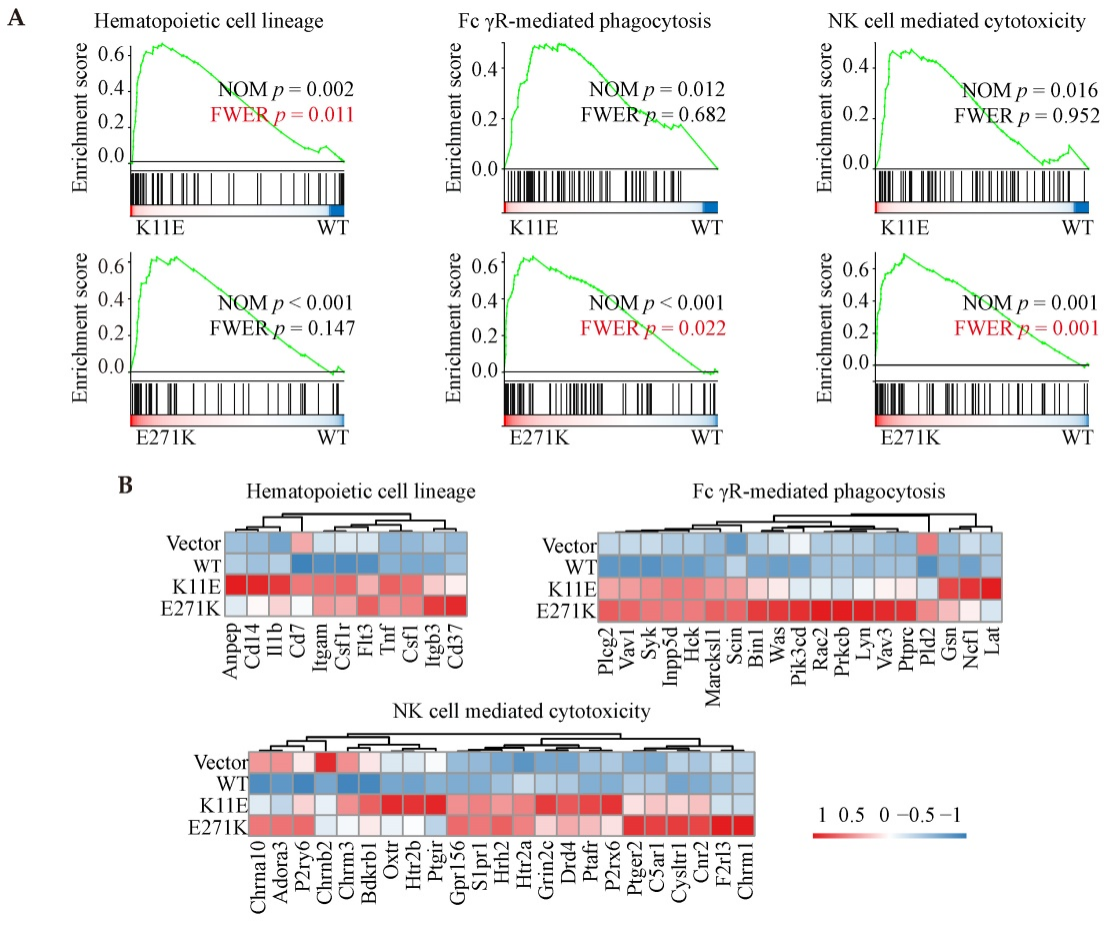
** GSEA enrichment analyses based on RNA-seq. (A)** Three immune-related pathways with *p* value corrected by Bonferonni test. **(B)** The heatmap analyses of gene in above three pathways.

In total, we identified 564, 1275, and 1007 DEGs in the three comparisons (Vector *vs.* WT, K11E *vs.* WT, and E271K *vs.* WT, respectively). Contrasted with the WT group, there were 257 genes up-regulated and 307 genes down-regulated in the Vector group, 546 genes up-regulated and 729 genes down-regulated in the K11E group, as well as 863 genes up-regulated and 144 genes down-regulated in the E271K group (Figure 5A). To guarantee the contrast, we selected 71 overlapped DEGs from our datasets (Figure 5B). The KEGG pathway enrichment (Figure 5C, 5D) and Go term enrichment (Figure 5E, 5F) based on the 71 DEGs indicated there was no significant difference between WT group and other group. However, GSEA enrichment showed that there were 25 pathways between K11E group and WT group and there were 68 pathways between E271K group and WT group. There were 15 overlapping pathways between the two contrasts (Table S2), where 8 pathways were related with immune regulation [hematopoietic cell lineage, Fc-γR-mediated phagocytosis, natural killer (NK) cell mediated cytotoxicity, leukocyte transendothelial migration, T helper 17 (Th17) cell differentiation, chemokine signaling pathway, C-type lectin receptor signaling pathway, and T cell receptor signaling pathway]. In addition, there were 3 pathways (hematopoietic cell lineage, Fc-γR-mediated phagocytosis and NK cell mediated cytotoxicity) (Figure 6A) still of significant difference after corrected by Bonferonni test. The heatmap analyses of gene expression in these 3 pathways (Figure 6B) showed that the expression of TNF and chemokines increased in mutant groups.

## Discussion

DDX24 was identified as a drug target in cancer therapeutics by screening of a shRNA library, which was agreed with the report by Li F in gastric cancer 19. Moreover, Yamauchi T, Shi D and their colleagues showed that the depletion of DDX24 might block p53 functions 11, 12, which indicated DDX24 seemed to be an oncogene. In previous study, the point mutations of *DDX24*, K11E and E271K, were related to vascular malformations disease, MOVLD. But whether these mutations work as “loss of function” or “gain of function” remains unknown. We found that the above two mutation sites were highly conserved in different species. It was generally believed mutations in highly conserved regions would lead to inability to survive or functional subversion. Many members of DDX family were reported to show decreased alteration in enzyme activity and function due to mutations in highly conserved regions. For example, DDX5 (Dbp5/DDX19B) shuttled between the nucleus and the cytoplasm to mediate the export of messenger ribonucleoprotein (mRNP). L12A at the N-terminal caused defection of mRNP export, which led to the failing growth of corresponding strain 20. Moreover, the point mutation in conserved residues of the ATPase active sites of DbpA in Escherichia coli (*E. coli*) reduced its ATPase activity and helicase activity 21. In other DExD/H helicases, such as Prp16, Prp22, Prp43 and eIF-4A, K53 or R331 mutations can lead to be lethal 22-26. We tried to purify recombinant wild-type DDX24 or mutant DDX24 proteins from *E. coli* or CHO cell lines to detect their activity of RNA helicase or ATPase, but all failed due to the instability of DDX24 itself *in vitro*. Analogous to other DDX, whether K11E or E271K DDX24 would lose its enzyme activity is still worthy to study further.

Recent advances highlighted the nucleolus, as an important membrane-less organelle, usually formed via phase separation 27-29. Our data showed DDX24 was co-localized with Npm1, a marker in the granular compartment 30 and a key driver of nucleolar phase separation 31, 32, which indicated that DDX24 distributed in nucleolus and participated in the composition of nucleolus. However, the specific and detailed compositions of nucleolus are not clear yet; previous study showed that the nucleolus consisted of three sub-structures including the fiber center, the dense fiber component and the granular components 33. A range of studies have characterized that the nucleolus played a significant role in genomic stability, stress response, DNA repair and recombination, transcription regulation, telomere maintenance, ribosome biogenesis and other essential cellular processes, which were closely related to cancer development 34, 35. In addition, nucleolar size was one of important parameters to estimate cell growth and proliferative intensity; meanwhile it has been used in some cancers as a predictive and prognostic biomarker for chemotherapy and clinical outcomes 36-38. Our IF and IHC staining revealed that compared with cells expressing wild-type DDX24, the number of droplets in K11E-DDX24 or E271K-DDX24 cells was significantly reduced, which implied that these mutations altered the quantity of nucleolus. Indeed, the heteroligomer state of native *E. coli* recombination human DDX24 has been studied in our institute by Dr. Shoudeng Chen, who has observed dimer or tetramer of DDX24 in solution (data not yet published). We also searched the literature, and found recent study regarding the eukaryotic expression of DDX24 with heterooligomer 39. Our existing study provided evidence that DDX24 formed heteroligomer with the mutated DDX24 proteins, therefore “dominant negatives” may be the underline mechanism that these mutations in cell and in tumor bearing mouse changed the oncogenicity of DDX24. As Lasry I reported, the alterational location of target protein in cell loss of zinc transport activity of the WT ZnT-2 due to homodimerization acted as dominant negatives 40. Nevertheless, the specific mechanism behind this alteration still remains unknown and is needed to do more explore.

Proliferation assays in human tumor cells showed that the expression of K11E or E271K DDX24 may inhibit relate cell proliferation in tumors with high expression of DDX24 or bad prognosis of DDX24, but not in tumors with low expression of DDX24 or none-risk prognostic of DDX24. Although we did not knockdown or knockout the endogenous expression of DDX24 in above human tumor cells, MHCC-97L, KYSE410 and HeLa cells transfected with mutant *DDX24* still exhibited proliferation suppression, which implied these mutations have lost these roles in cellular proliferation. Moreover, proliferation assays in CHO cells along with further *in vivo* tumor implantation studies also suggested “loss of function of cell proliferation” for these two mutations.

To further investigate the *in vivo* oncogenicity of DDX24 mutations, ^18^F-FDG PET/CT was applied to investigate the imaging quantification of DDX24 expressed tumors. SUV was widely used for PET imaging quantification 41. However, many factors such as the tumor heterogeneity, uptake kinetics and post injection time can influence the feedback of SUV 42, 43, which appeared less specific for tumor proliferation. Thus, the K_i_ value, a quantitative measure of the influx rate constant of ^18^F-FDG derived from the dynamic PET scans was calculated 17. In this study, tumors of each group were size-matched. From this quantitative analysis, both SUV and K_i_ value of WT-DDX24 group were higher than that of the vector controls, which convincingly implied that WT-DDX24 accelerated the glucose utilization rate and enhanced the tumor proliferation rate. The majority of mutant groups (K11E and E271K) showed less glucose utilization than that of WT groups, suggested “loss of function” of *in vivo* oncogenicity for these two mutations of DDX24.

Moreover, RNA-seq from the tumors formed by mutant DDX24 showed that at least 8 immune-related pathways (hematopoietic cell lineage, Fc-γR-mediated phagocytosis, NK cell mediated cytotoxicity, leukocyte transendothelial migration, Th17 cell differentiation, chemokine signaling pathway, C-type lectin receptor signaling pathway, and T cell receptor signaling pathway) were altered in comparison between wild-type group and mutant groups. In addition, several immune-correlated factors such as TNF and chemokines in mutant tissues were highly expressed. Even though nude mice do not have conventional CD4^+^ and CD8^+^ T cells or enough effective B cells to have the signaling altered, nude mice possessed precursor cells for the T compartment of the lymphoid system as previous Loor F reported 44. Unconventional T cells including natural killer T cells, mucosal-associated invariant T cells and γδ T cell were thymus-independent and made up ~10% of circulating T cells 45. Given nude mice were not defective in the responder cells of NK cell lineage 46, we speculated that K11E-DDX24 or E271K-DDX24 may enhance non-specific immune killing to inhibit cell proliferation and tumor growth.

In summary, our present work verified K11E or E271K mutation led to “loss-of-function” of DDX24 in cell proliferation and tumor bearing mice by using a CHO cell model. The tumor suppression induced by K11E or E271K mutation of DDX24 is expected to provide a novel idea or direction for tumors prevention and treatment, especially for those tumors in which DDX24 plays an oncogenic role in proliferation.

## Supplementary Material

Supplementary materials and methods, figures and tables.

## Abbreviations

DDX: DEAD-box RNA helicase
MOVLD: multi-organ venous lymphatic malformation syndrome
CHO: Chinese hamster ovary cell
IF: immunofluorescence
*vs.*: verses
LIHC: liver hepatocellular carcinoma
ESCA: esophageal carcinoma
CESC: cervical squamous cell carcinoma and endocervical adenocarcinoma
PRAD: prostate adenocarcinoma
UCEC: uterine corpus endometrial carcinoma
IHC: immunohistochemistry
PET: positron emission tomography
SUV: standardized uptake value
RNA-Seq: RNA-sequencing
DEG: differentially expressed genes
KEGG: Kyoto Encyclopedia of Genes and Genomes
GO: Gene Ontology
GSEA: Gene Set Enrichment Analyses
NK cell: natural killer cell
Th17 cell: T helper 17 cell
*E. coli*: Escherichia coli
