# Supplementary material for: Loss-of-function Mutations K11E or E271K Lead to Novel Tumor Suppression, Implicate Nucleolar Helicase DDX24 Oncogenicity
Source: Int J Med Sci. 2022 Mar 14;19(3):596–608. doi: 10.7150/ijms.67840 (PMC8964322; doi:10.7150/ijms.67840)
Supplement: Supplementary file 1 — Supplementary materials and methods, figures and tables. [file ijmsv19p0596s1.pdf]

Supplementary File

1. Materials and methods

1.1 Knocking down *DDX24* in CHO-WT-DDX24 cells

CHO-WT-DDX24 cells were transfected with DDX24-shRNA (HSH015368-33-CU6, Funeng Gene, Guangzhou), and the cells transfected with DDX24-NC were the negative control. Successfully transfected cells were screened by 2 μg/mL puromycin (P8230, Solarbio). Finally, the monoclonal stable transfected strains (CHO-WT-DDX24-ShRNA and CHO-WT-DDX24-NC) were screened out. After verificating through qPCR and Western blot, cell proliferation assay and colony formation assay were performed.

1.2 Bioinformatics Analysis (gene expression, survival and genetic alteration analysis)

To observe the expression diversity of *DDX24* between different tumors and corresponding normal tissues of the cancer genome atlas (TCGA) project, we entered DDX24 in the "Expression DIY_Box Plot" module under default parameters (|Log2FC| cutoff = 1, *p* value cutoff = 0.01, and "Match TCGA normal and GTEx data") of Gene Expression Profiling Interactive Analysis_version 2 (GEPIA2) (http://gepia2.cancer-pku.cn/#analysis) [1]. Then we obtained survival plot of liver hepatocellular carcinoma (LIHC) through "Survival Analysis" module of GEPIA2 under the settings of significance Level = 0.05 without p-value adjustment, cutoff-high (50%) and cutoff-low (50%). Additionally, we explored OS analyses of cervical squamous cell carcinoma and endocervical adenocarcinoma (CESC) and esophageal carcinoma (ESCA) across GEO datasets by using "mRNA RNA-seq_Pan-cancer" module of Kaplan-Meier Plotter website (http://kmplot.com/analysis/) [2] through setting "Auto select best cutoff" to split every cancer case into two groups. To search for the genetic alteration characteristics of *DDX24*, we used the "Query_Quick select_TCGA PanCancer Atlas Studies" module of the cBioPortal website (https://www.cbioportal.org/) [3, 4]. We obtained the mutation type and mutation site of *DDX24* in all TCGA tumors through "Cancer Types Summary" module.

2. Supplementary tables

Table S1. Sequence of primers used in construction of plasmids.

Gene	Sequence 5'~3'
WT-DDX24	**F** GAAGATCTGCCACCATGAAGTTGAAGGACACAAA
	**R** CCCAAGCTTGTGATGGTGATGGTGATGGCCCTGAAAATACAGGT
K11E-DDX24	**F** AAGGACACAAAATCAAGGCCAGAGCAGTCAAGCTGTGGCAAA
	**R** TTTGCCACAGCTTGACTGCTCTGGCCTTGATTTTGTGTCCTT
E271K-DDX24	**F** CTCCTCCAAGTAACACCAAAGCACCACCTGGAGAGA
	**R** TCTCTCCAGGTGGTGCTTTGGTGTTACTTGGAGGAG

[1] The underline sequences were BglII site (F) and HindIII (R).

Table S2. GSEA of RNA sequencing from solid tumor derived from Balb/c-nu mice.

Gene Set	Size		NES		NOM *p*-val		FDR *q*-val		FWER *p*-val	
	K-W	E-W	K-W	E-W	K-W	E-W	K-W	E-W	K-W	E-W
Cytokine-cytokine receptor interaction	163	162	1.598	1.457	0.001	0.003	0.300	0.083	0.890	0.990
Chemokine signaling pathway	134	134	1.534	1.721	0.011	0.000	0.318	0.011	0.978	0.124
NF-κB signaling pathway	78	78	1.636	1.452	0.005	0.014	0.243	0.086	0.806	0.993
Neuroactive ligand-receptor interaction	145	143	1.493	1.491	0.013	0.001	0.272	0.066	0.991	0.966
Apelin signaling pathway	119	119	1.463	1.387	0.015	0.018	0.310	0.123	0.997	1.000
C-type lectin receptor signaling pathway	90	90	1.409	1.419	0.040	0.022	0.353	0.105	1.000	0.998
Hematopoietic cell lineage	49	49	1.984	1.707	0.002	0.000	0.012	0.010	0.011	0.147
Natural killer cell mediated cytotoxicity	67	67	1.566	1.940	0.016	0.000	0.316	0.000	0.952	0.001
Th17 cell differentiation	79	79	1.493	1.567	0.019	0.002	0.284	0.038	0.991	0.744
T cell receptor signaling pathway	86	86	1.470	1.733	0.027	0.000	0.308	0.011	0.994	0.106
Fc γR-mediated phagocytosis	73	73	1.672	1.804	0.005	0.000	0.241	0.005	0.682	0.022
Leukocyte transendothelial migration	88	88	1.527	1.712	0.012	0.000	0.257	0.010	0.981	0.134
Insulin secretion	63	63	1.591	1.759	0.012	0.000	0.280	0.010	0.897	0.068
Gastric acid secretion	58	58	1.542	1.665	0.021	0.001	0.352	0.018	0.975	0.294
Carbohydrate digestion and absorption	27	27	1.656	1.465	0.016	0.037	0.235	0.078	0.737	0.987

[1] NES, Normal Enrichment score; NOM *p*-val, *p* value; FDR *q*-val, *q* value; FWER *p*-val, *p* value corrected by Bonferonni.

[2] K-W, contrast bewteen group K11E and group WT; E-W, contrast bewteen group E271K and group WT.

[3] Red font: the *p* value, corrected by Bonferonni, was significant statistically.

3. Supplementary figures

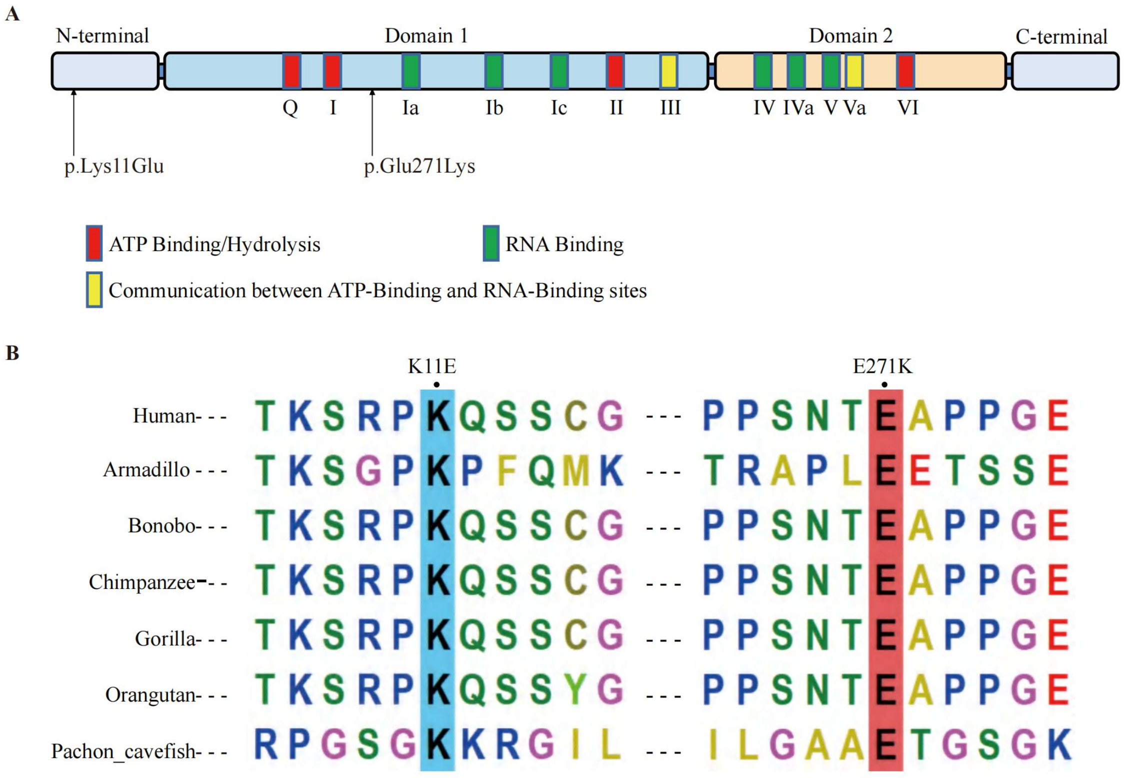

Supplementary Figure 1. Conservative analyses of 11aa and 271aa sites of DDX24. (A) Structure of the Dead Box RNA Helicases family. **(B)** Sequence alignment of DDX24 across distinct species.

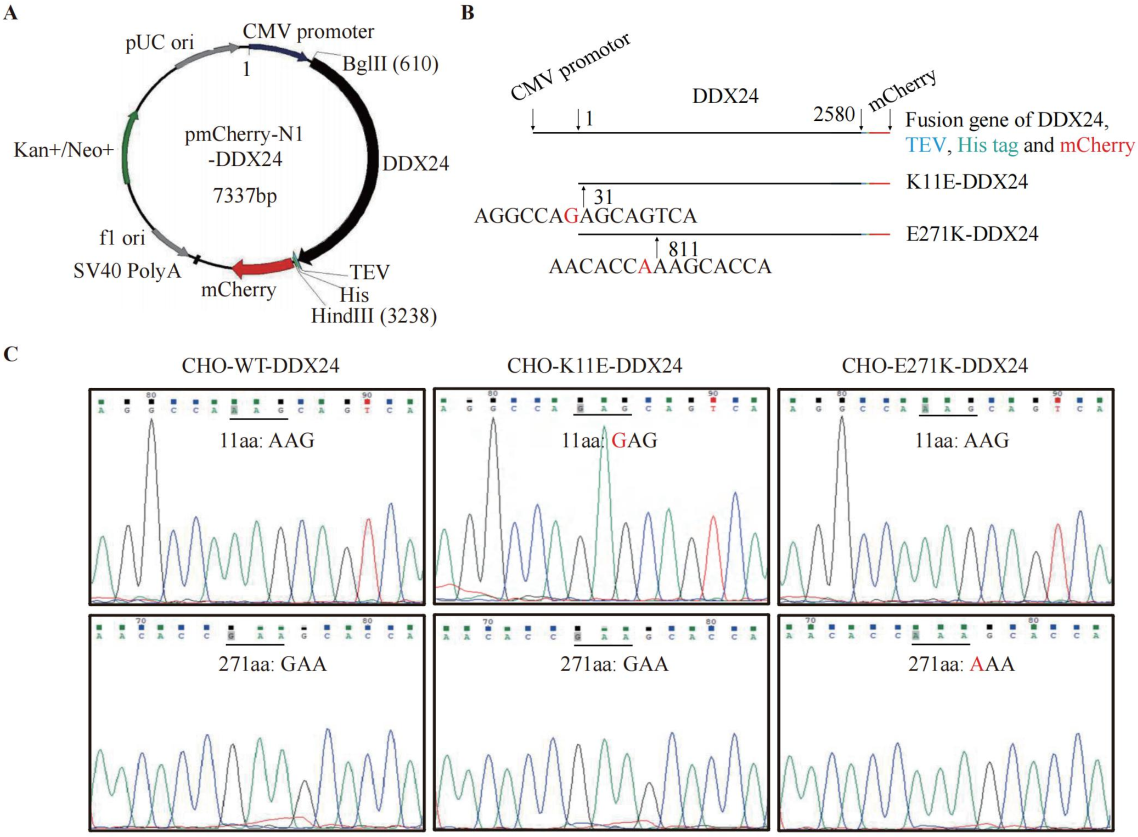

Supplementary Figure 2. Construction of DDX24 plasmids and sequencing of *DDX24* in constructed CHO cell lines. (A) The schematic diagram of plasmid constructed with pmCherry-N1 as the carrier. **(B)** Two kinds of point mutations of *DDX24*. **(C)** Sequence of CHO-WT/K11E/E271K-DDX24 cells at codon 11st and 271st, respectively.

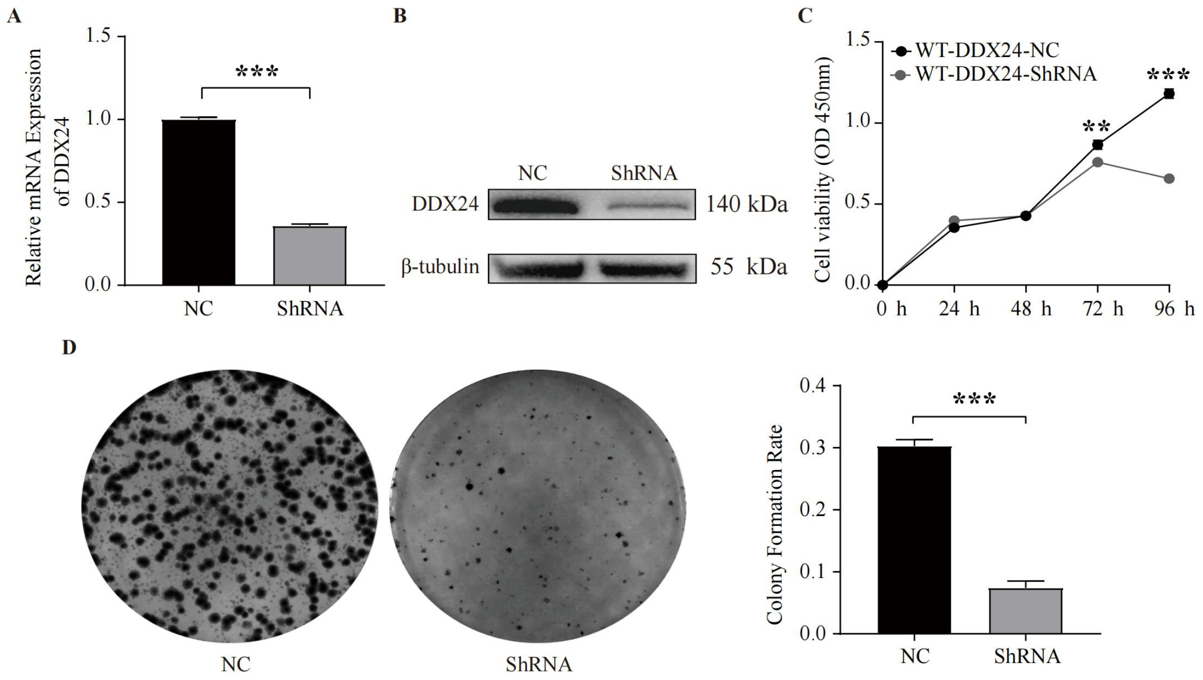

Supplementary Figure 3. *DDX24* knockdown in CHO-WT-DDX24. Verification of *DDX24* Knockdown through qPCR **(A)** and Western Blot **(B)**. Cell proliferation assay **(C)** and colony formation assay **(D)** were performed. *P* values were calculated with unpaired T test. **, $p < 0.01$, $p = 0.0034$; ***, $p < 0.001$.

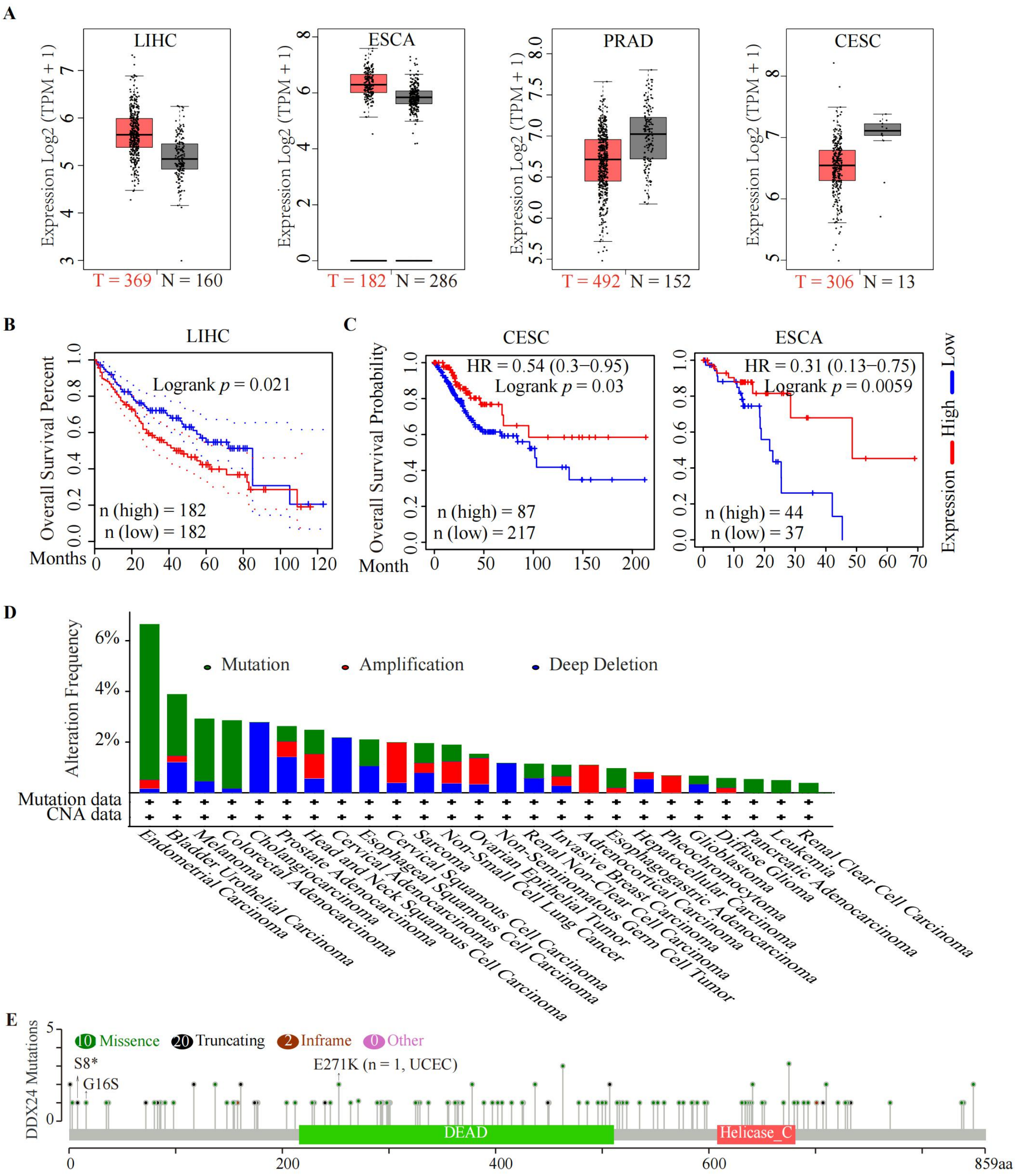

Supplementary Figure 4. DDX24 in human tumor tissues. The gene expression analyses of *DDX24* in LIHC, ESCA, PRAD and CESC from GEPIA2 **(A)**. Overall survival analyses of different tumors were obtained by using GEPIA2 **(B)** and Kaplan-Meier Plotter **(C)**. *DDX24* mutation characteristics **(D)** and mutation sites **(E)** in TCGA tumors from cBioPortal website.

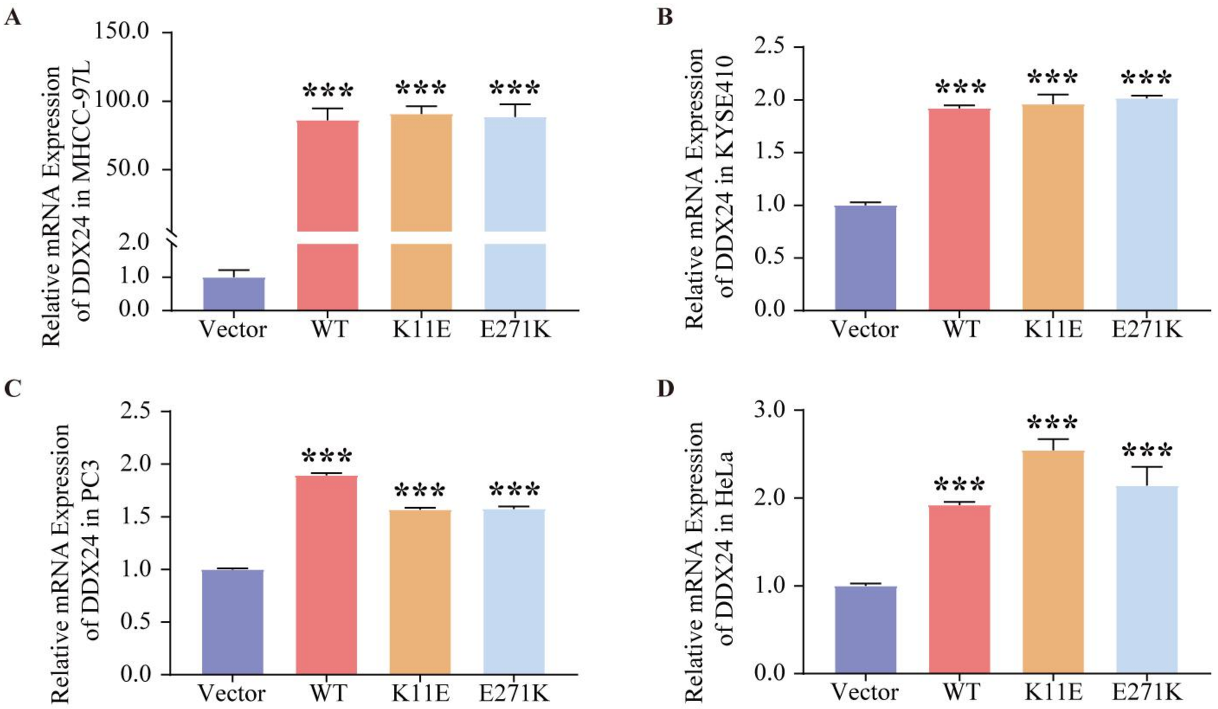

Supplementary Figure 5. Detection of DDX24 overexpression in tumor cells.

Relative mRNA levels of *DDX24* in constructed MHCC-97L **(A)**, KYSE410 **(B)**, PC3 **(C)** and HeLa **(D)** cell lines were detected by qPCR. Cells transfected with pmCherry-N1 empty plasmid (Vector) were regarded as the negative control. *P* values were calculated with Tukey multiple comparisons test. ***, $p < 0.001$.

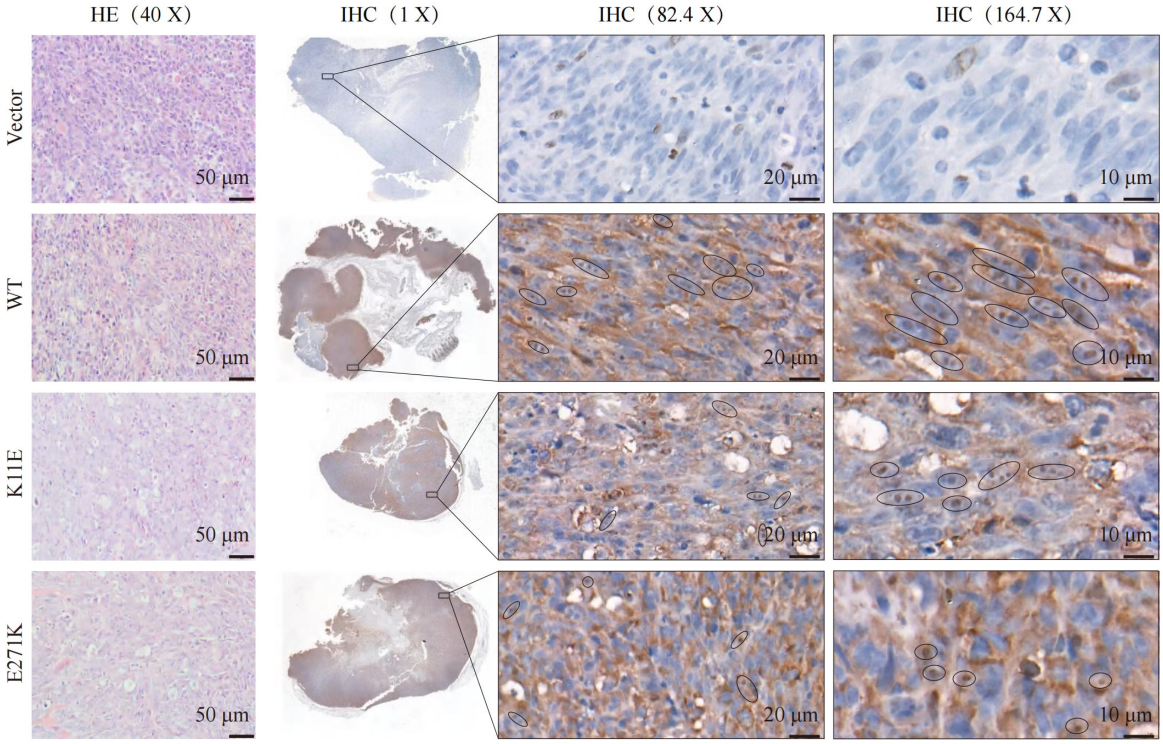

Supplementary Figure 6. Slices staining of tumor tissues. Immunohistochemical (IHC) staining confirmed the expression of DDX24 in tumors. Inside the black circle were nucleoli dyed deeply.

4. Supplementary references

1. Tang Z, Kang B, Li C, et al. GEPIA2: an enhanced web server for large-scale expression profiling and interactive analysis. Nucleic Acids Res. 2019;47(W1):W556-W560.
2. Nagy A, Lanczky A, Menyhart O, et al. Validation of miRNA prognostic power in hepatocellular carcinoma using expression data of independent datasets. Sci Rep. 2018;8(1):9227.
3. Cerami E, Gao J, Dogrusoz U, et al. The cBio cancer genomics portal: an open platform for exploring multidimensional cancer genomics data. Cancer Discov. 2012;2(5):401-404.
4. Gao J, Aksoy B, Dogrusoz U, et al. Integrative analysis of complex cancer genomics and clinical profiles using the cBioPortal. Sci Signal. 2013;6(269):pl1.
